# Fast pain relief in exercise-induced acute musculoskeletal pain by turmeric-boswellia formulation: A randomized placebo-controlled double-blinded multicentre study

**DOI:** 10.1097/MD.0000000000030144

**Published:** 2022-09-02

**Authors:** Girish H. Rudrappa, Meghana Murthy, Santosh Saklecha, Sanjeev Kumar Kare, Ajay Gupta, Indraneel Basu

**Affiliations:** a Department of Orthopedics, Sapthagiri Institute of Medical Sciences and Research Centre, Bangalore, India; b Vagus Hospital, Bangalore, India; c Santosh Hospital, Bangalore, India; d Government Medical College & General Hospital, Srikakulam, India; e Nirmal Hospital, Jhansi, India; f Sudbhawana Hospital, Varanasi, India.

**Keywords:** acute pain, analgesic, exercise, musculoskeletal pain, pain relief

## Abstract

Plant extracts with analgesic properties are seldom considered for treatment of acute musculoskeletal pain due to delay in onset of analgesia. Turmeric (*Curcuma longa*) and boswellia (*Boswellia serrata*) extracts are well-studied anti-inflammatory compounds gaining in popularity and used as an alternative to conventional treatments for musculoskeletal pain. This study analyzed the analgesic effect of a formulation of turmeric and boswellia extracts in sesame oil (Rhuleave-K, TBF) in reducing exercise-induced acute musculoskeletal pain in healthy participants.

**Methods:**

In this randomized double-blinded placebo-controlled, single-dose, single-day, multicentre study, a total of 232 participants (TBF n = 116; placebo n = 116) having moderate-to-severe exercise-induced acute musculoskeletal pain were randomized in an allocation concealed 1:1 ratio to receive a single dose of 1000 mg of TBF or placebo. The outcome measures were numerical rating scale (NRS), categorical pain relief scale (PRS), onset of analgesia, and short form of McGill questionnaire (SF-MPQ). NRS and PRS were measured from predose to every 30 minutes interval of postdose up to 6 hours at rest, with movement and applying pressure on the affected part. The onset of analgesia was measured from the time of dosage and censored at 6 hours of postdose. The sum of pain intensity difference (SPID6) and total pain relief (TOTPAR6) at 6 hours was, respectively, analyzed from NRS and PRS scores.

**Results:**

TBF showed a significant reduction in pain intensity (SPID6_rest_) with 97.85% improvement in cumulative responder analysis compared with 2.46% in placebo. The onset of pain relief was fast and highly significant in the TBF group with 99% of participants having a mean perceptible pain relief at 68.5 minutes (95% confidence interval, 59.5–77.4) and 96% of participants having a mean meaningful pain relief at 191.6 minutes (95% confidence interval, 176.7–206.4) compared to the placebo group. Highly significant and continuous improvement in pain relief was observed in the TBF group with 93% of participants having ≥ 50% of maximum TOTPAR6 with a number needed to treat of 1.1 at rest.

**Conclusion:**

Exercise-induced acute musculoskeletal pain can be effectively relieved by TBF (Rhuleave-K) in about 3 hours signifying its strong analgesic activity.

## 1. Introduction

Musculoskeletal pain is very common in everyday life, especially when the body performs tasks, oftentimes in awkward positions or doing infrequent activities. Physical exercise has beneficial effects but strenuous and eccentric exercise comes with a cost and injuries are very common. It leads to injuries or inflammatory conditions that cause pain in the body’s joints, ligaments, muscles, nerves, tendons, and structures that support the limbs, neck, and back. Pain medicine has evolved over recent years into a large specialty area, being recognized as its discipline. Management of pain continues to be one of the most commonly encountered clinical situations for practitioners and remains a major challenge for researchers and clinicians.^[1]^

The demand for oral analgesics dominates both the prescription and nonprescription drug markets. Over-the-counter analgesics like ibuprofen showed no increase in skeletal muscle fractional synthesis rate postexercise^[2]^ and the normal increase in prostaglandin synthesis was suppressed.^[3]^ A subsequent investigation by this group concluded that the cyclooxygenase-1 (COX-1) enzyme is the isoform responsible for the COX-mediated increase in protein synthesis, stressing the importance of this enzyme in human muscle and the potential negative effect of COX-1 specific inhibition.^[4]^ The use of non-steroidal anti-inflammatory drugs to alleviate moderate pain after acute or exercise-induced muscle injury requires further studies in light of the above findings.^[5]^ There is a great prospect in considering alternative for pain management. Natural remedies are preferred as they are safe and effective at treating inflammation and pain and have a history of being used for millennia in traditional medicine.^[6]^

Herbal preparations have been used from ancient times to obtain effective pain relief.^[7,8]^ Turmeric and boswellia are well-studied anti-inflammatory compounds gaining in popularity and being used as an adjunct to, but also as an alternative to, conventional treatments for musculoskeletal pain. Curcumin and boswellic acids, the active ingredients of turmeric rhizomes and *Boswellia serrata* gum resin are known to inhibit the nuclear factor κB signaling pathway, which is directly involved in the inflammatory processes. Dietary supplements with *Curcuma longa* and *B serrata* considered the most effective compounds for pain reduction in osteoarthritis at short-term.^[9]^ Transient receptor potential channel, vanilloid subfamily member 1 (TRPV1) antagonists are currently undergoing clinical trials for indications related to pain since it makes the neurons involved in pain inactive. Another approach in pain treatment is to use targeted neurotoxins to cause neuronal death.^[10]^ Arachidonic acid derivatives that are present in pain pathway also activate TRPV1.^[11]^ All these activations make the neurons responsible for pain transmission inactive.

Though herbal treatments are popular for managing chronic pain, the slow onset of action and lack of rigorous clinical validation has limited their use for acute pain. A single-center, active-controlled, open-label pilot study was conducted with turmeric-boswellia formulation (TBF) (1000 mg/d for 7 days) for acute musculoskeletal pain at resting position and the results demonstrated similar pain relief effect similar to acetaminophen^[12]^ most likely due to its effect in modulating multiple pathways (inactivation of neurons/anti-inflammatory) for quick relief of pain. The present double-blind placebo-controlled multicentre study was planned to understand the effect of TBF (Rhuleave-K) on exercise-induced moderate-to-severe acute musculoskeletal pain and confirm the earlier study findings on a statistically powered sample size. The objective of the study was to evaluate the efficacy of a single dose of 1000 mg of TBF in reducing exercise-induced acute musculoskeletal pain and analyze the onset of pain relief and improvement in pain intensity (PI) at resting position, with movement and applying pressure on the affected part.

## 2. Methods

### 2.1. Study design

This randomized placebo-controlled double-blinded multicentre study enrolled 232 healthy participants with exercise-induced acute musculoskeletal pain. The study employed a parallel interventional model with an allocation (TBF: placebo) ratio of 1:1 and a male to female ratio of 1:1. The study protocol was approved by the Institutional Ethics Committee of each participating hospital and no amendments to the accepted protocol were done after starting the study. The study was conducted at 6 sites in India at geographically different regions from August 5, 2020 to October 21, 2020 following their institutional governance guidelines, principles of the Declaration of Helsinki, and the International Conference on Harmonization–Good Clinical Practice guidelines and was registered in Clinical trial registry of India (CTRI/2020/06/025601, Registered on June 4, 2020). The study protocol was reviewed and approved by Rajalakshmi Hospital Institutional Ethics Committee (RHIEC), Vidyaranyapura, Bangalore (Approval number RH/IEC/AP-056/2020 dated March 21, 2020), Vagus Institutional Ethics Committee, Malleshwaram, Bangalore (Approval dated May 14, 2020), Santhosh Hospital Institutional Ethics Committee, Promenade road, Bangalore (Approval number SHIEC/CC/MAR_2020/03 dated March 19, 2020), Institutional Ethics Committee Government Medical College and Government General Hospital, Srikakulam, Andhra Pradesh (Approval dated September 12, 2020), Institutional Ethics Committee Nirmal Hospital, Jhansi, Uttar Pradesh (Approval dated September 3, 2020) and Sudbhawana Hospital Ethics Committee, Lanka, Varanasi (Approval dated August 29, 2020).

### 2.2. Study procedure

The study population came from the outpatient department of participating centers. Participants who were ready to give voluntary informed consent and who meets the study inclusion and exclusion criteria were selected. The participants were asked for the specific history of pain like date of occurrence, time of onset, duration of pain, part of the body affected, history of similar pain in the past during exercise (<24, 24–48, and >48 hours), its severity, duration of pain, any treatment taken, etc.

### 2.3. Study inclusion/exclusion criteria

The study included healthy male and female participants aged 18 to 65 years with exercise-induced acute musculoskeletal pain with a resting numerical rating scale (NRS) of 5 or above on a 0 to 10 scale, which occurred within 24 hours before presenting at the site. Exercise-induced musculoskeletal injuries, myalgia, neck pain, limb pain, low back pain, joint pain, widespread musculoskeletal pain, painful uncomplicated acute soft tissue injury of the upper or lower extremity, including acute injuries of ligaments, tendons, or muscles (including Grade 1 sprain or strain) that occurred within 24 hours and do not require admittance to the hospital were included in the study. Most of the participants were regular gym goers and the rest did home exercises. Participants with acute muscle spasms requiring parenteral therapy or surgery; hospital admission for management of painful acute soft tissue injury of the upper or lower extremity, including acute injuries of ligaments, tendons, or muscles, or Grade 2 and 3 sprain or strain; or with history of osteoarthritis or rheumatoid arthritis, etc., or consuming any products for pain and inflammation in the week before the study were excluded.

### 2.4. Intervention and dosing

The participants who met all the inclusion and exclusion criteria were randomized to receive either the test intervention TBF (Rhuleave-K) 1000 mg (500 mg × 2 softgels) containing 266 mg curcuminoids and 10 mg acetyl keto-boswellic acid (AKBA) or comparator intervention of matching placebo 1000 mg (500 mg × 2 softgels) manufactured by Arjuna Natural Pvt. Ltd, Aluva, India. TBF contains turmeric (*C longa* L.) extract, *B serrata* extract, and black sesame (*Sesamum indicum*) seed oil. Turmeric rhizomes were extracted using ethyl acetate and were standardized to contain 26.6% curcuminoids. *B serrata* gum resin was extracted using ethyl acetate and was standardized to contain 1% AKBA and the actives of *C longa* and *B serrata* were solubilized uniformly in sesame seed oil using proprietary technology. TBF and placebo were encapsulated in size “0” vegetarian reddish-brown colored soft gel capsules. The matching placebo was a blend of maltodextrin in an excipient base of polysorbate-80, propylene glycol, and polyethylene glycol-400 in quantity well within the acceptable daily intake.

### 2.5. Randomization, blinding, and unblinding

The subjects were stratified into male and female in 1:1 ratio and randomized to TBF and placebo groups in 1: 1 ratio in all 6 sites taken together using the software WinPepi version 11.65 (2016) by an independent statistician. The allocation was concealed using opaque bottles and alphanumeric codes such that the study products were identified only by their allocation codes. The allocation concealed randomization code list was given to the pharmacist for the dispensation of study materials. The randomization schedule, as well as the study materials, were under the restricted access of the pharmacist to prevent selection bias and were serially administered. No other study staff was involved in the dispensing process. The investigators and the subjects were blinded using a placebo with similar size, color, packaging, and labeling. All staff engaged with the study were blinded to the identity of the treatments. Sealed, opaque envelopes containing package inserts with identity of the study products were given to the pharmacist. In emergency cases that require unblinding, the investigator would intimate the pharmacist and the pharmacist would provide the envelope of that participant to the investigator. The investigator would also inform the sponsor regarding the need to unblind and also document the events.

### 2.6. Outcome assessments

The primary outcome was a change in the sum of pain intensity difference (PID) at 6 hours at rest (SPID6_rest_) calculated from NRS.^[13]^ The secondary outcomes were the time to perceptible pain relief (PPR) and time to meaningful pain relief (MPR) using the double-stopwatch method (onset of analgesia)^[14]^; SPID6 at movement (SPID6_move_) and pressure (SPID6_pres_); total pain relief at 6 hours at rest (TOTPAR6_rest_), movement (TOTPAR6_move_) and pressure (TOTPAR6_pres_) using pain relief scale (PRS)^[15]^ and the change in the quality of pain using short form of McGill pain questionnaire (SF-MPQ).^[16]^ The derived outcomes from NRS scores were PID, Time-weighted sum PID (SPID) at 180 and 360 minutes; Area under the PID curve at 180 and 360 minutes^[13]^, and cumulative proportion of responder’s analysis (CPRA)^[17]^ from the area under the curve (AUC) for rest, movement, and pressure. The number needed to treat (NNT) was calculated as an additional outcome of the PRS score for rest, movement, and pressure.^[18]^ Time points and the schedule of activities are summarized in (Table S1, Supplementary Digital Content 1, http://links.lww.com/MD/H112). Since the study was of 6 hours, a detailed safety assessment was not conducted. Safety assessment was done based on physical examination, vital signs, and treatment-emergent adverse events such as hypersensitivity or patient-reported events.

#### 1.2.6. Numerical pain rating scale.

The NRS is an 11-point scale, in which 0 represents “no pain” and 10 represents the worst pain possible.^[19]^ The participants were asked to rate their pain intensity as a number from 0 to 10. The NRS was taken at rest, on movement of the affected part, and on applying pressure to the affected part at screening, and those participants who have 5 or above score for NRS (rest) were enrolled in the study. After dosing, the pain intensity rating of the participants was taken at rest, movement, and pressure on every 30 minutes up to 6 hours postdose for calculating SPID 6 hours.

#### 2.2.6. Pain relief scale.

The PRS is a categorical scale having a positive progression from “No relief”, “A little relief”, “Some relief”, “A lot of relief” to “Complete relief” (coded 0 to 4). TOTPAR is the area under the time-analgesic effect curve for a given time.^[15]^ After dosing, the participants rated the pain relief obtained using PRS at rest (PRS_rest_), on movement of the affected part (PRS_move_), and on applying pressure to the affected part (PRS_pres_) every 30 minutes up to 6 hours for the assessment of TOTPAR 6 hours.

#### 3.2.6. Onset of analgesia.

The onset of analgesia was taken using the “double stopwatch” method. After dosing, the 2 stopwatches were started simultaneously by the study coordinator and were given to the participant. The first stopwatch was stopped when the participant reports the first PPR. The second stopwatch was stopped when the participant felt complete pain relief called MPR. The time to PPR and MPR was recorded in hours and minutes, and the seconds were rounded to the next minute if above 30 seconds. If PPR/MPR was not reached within 6 hours, it was censored at that time point.^[14,20]^

#### 4.2.6. Short form of McGill pain questionnaire.

The MPQ allows the participant to describe the quality (affective domain) and intensity (sensory domain) of the pain and were answered at baseline and at the end of the study. Participants also rated their present pain intensity (PPI) on a 0 to 5 scale. Pain intensity was assessed on a 0 to 100 mm horizontal VAS, anchored by no pain (score of 0) and worst possible pain (score of 100).^[16]^

### 2.7. Statistical analysis

#### 1.2.7. Sample size estimation.

A sample size of 219 was estimated to achieve 80% power to detect a mean difference of 0.450 between the 2 groups in the primary efficacy variable NRS at rest, based on a standard deviation (SD) of the response variable 1.250 and intraclass correlation of 0.100^[21]^ with a significance level of 0.05 obtained from a mixed-effects model fit without the treatment-by-center interaction. A 5% dropout was estimated which equals 12 in absolute numbers, and hence, a total of 231 participants were needed. To have an equal number between the 2 groups, a total of 232 was taken as the sample size for the study. In the absence of previous data with placebo, SD was calculated as a range divided by 4.^[22]^ The range is the maximum − minimum of the primary efficacy variable (NRS at rest), which is restricted to 5 by inclusion criteria. PASS2020 (NCSS LLC, Kaysville, Utah, USA) software was used.

#### 2.2.7. Statistical analytical methods.

Pain intensity was assessed over 6 hours following oral administration of a single dose of study medication administration, where pain intensity was measured using a numerical rating scale. Analysis of NRS-derived endpoints (PID, SPID, and AUC) was completed using linear mixed models for repeated measures, t-test, or Mann–Whitney test. SPID and AUC were calculated at 180 and 360 minutes.^[13]^ PID (change from baseline, PI_baseline_ − PI_time_) information was analyzed using a longitudinal mixed model for repeated measures with fixed effects for treatment, time, treatment-by-time interaction, and random effect for participants. Treatment differences from placebo were estimated from the least square mean (LSM) with 95% confidence intervals (CIs) and associated 2-sided *P*-values under a null hypothesis that no mean difference existed between the groups.

The CPRA by Farrar *et al*^[17]^ with 95% CI calculated using bootstrapping with 5000 iterations were used in this study. The CPRA graphs present the cumulative proportion of participants who achieved a specific response rate (or percentage) as an improvement from baseline, determined by levels of response from lowest to highest. In the current analysis, responders were defined as participants with improvement from baseline greater or equal to zero; nonresponders (participants with scores showing a change score from baseline <0) were not considered. All calculations were performed using NCSS2020 (NCSS LLC, Kaysville, Utah, USA) and R 3.6.3 (R Core Team, Vienna, Austria).

For the onset of analgesia, PPR and MPR were analyzed by Kaplan-Meier (KM) curve and compared using the log-rank test. The test statistic was compared with a χ² distribution with 1 degree of freedom. Median time to onset, restricted mean survival time (RMST), and restricted mean survival time lost (RMLT) were calculated from the KM analysis.^[23,24]^ Additionally, the proportional hazard assumption was checked graphically using Schoenfeld residual test and checked by observing survival curves.^[25]^ To detect a difference between survival probabilities between the group, log-rank test, Gehan generalized Wilcoxon procedure, and Tarone–Ware was used. The NRS score at rest was a significant covariate associated with both PPR and MPR in the Cox regression model.

TOTPAR between the groups was analyzed using the Mann–Whitney *U* test. The NNT was calculated for achieving at least 50% of maximum TOTPAR and significance was determined using the Wald Chi-square test. The proportional odds model (Hosmer and Lemeshow) was fitted in PRS_rest, move, pres_ using the *polr* function from the MASS package with R.

Wilcoxon signed-rank test was done in MPQ, VAS, and PPI for within-group analysis. Standardized response mean was calculated to detect clinically significant change in MPQ, VAS, and PPI. The number of participants experiencing treatment effect as measured by MPQ was calculated using the smallest detectable difference at 68% CI (SDD_68_). Relative reliability, association, and bias between pre- and postmeasurements in MPQ for both groups were measured using intraclass correlation coefficients (ICCs).

## 3. Results

**Figure 1.**
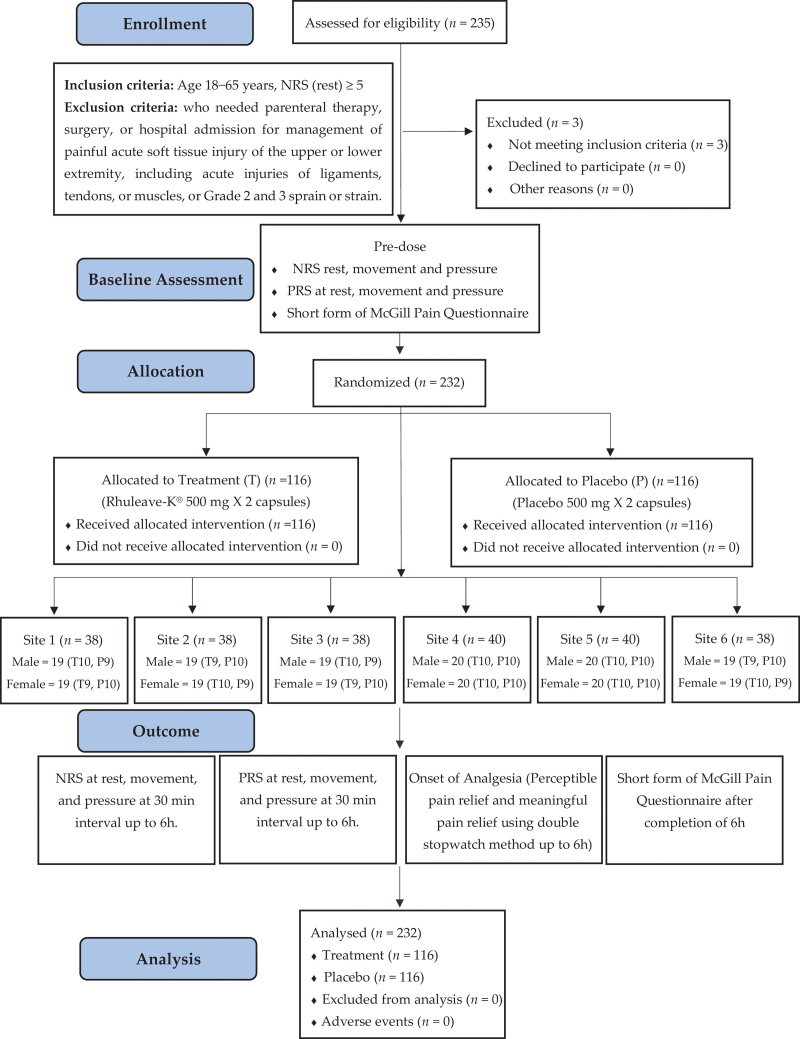
Study flow diagram.

In the study, 235 participants were screened and 232 were enrolled with 4 sites enrolling 38 each, and 2 sites enrolling 40 participants each. The study flow diagram is represented in Figure 1. There was no significant difference between the groups in the baseline parameters of NRS rest (*P* = .053), movement (*P* = .357), and pressure (*P* = .266). Demographics and baseline data of TBF and placebo groups are given in (Table S2, Supplementary Digital Content 2, http://links.lww.com/MD/H113). The participants enrolled had exercise-induced pain in lower back (n = 70), shoulder (n = 22), knee (n = 20), lower body (n = 22), and other types of pain (n = 98). This was a single-day single-dose study and all the study participants took the required dose of study medication and there was 100% treatment compliance. There were no adverse events reported in the study and no dropouts in this study.

### 3.1. NRS on rest, movement, and pressure

Time-weighted SPIDs and area under the curve of 180 and 360 minutes for NRS at rest, movement, and pressure showed significant difference (*P* < .001) between TBF and placebo and is given in Table 1. In the AUC responder analysis using NRS at rest, movement, and pressure, respectively, registered a difference of 95.39%, 93.52%, and 93.28% more than placebo responders at the end of 6 hours. Figures representing the CPRA are presented in (Figs. S1–S3, Supplementary Digital Content 3, http://links.lww.com/MD/H114). The LSM difference of TBF from placebo showed a statistically significant difference for all categories (NRS Rest, Movement & Pressure, *P* < .005) from 1 hour onwards till the end of the study at the specified time points. The NRS analysis showed that TBF reduced acute pain intensity, which was statistically and clinically significant. LSM PID of TBF from placebo and responder analysis for pain improvement for NRS (Rest, Movement, and Pressure) is detailed in Tables 2 and 3.

**Table 1:** Time-weighted sum of pain intensity differences and area under the curve of 180 and 360 min for NRS (rest, movement, and pressure).

Parameter	Group	NRS rest			NRS movement			NRS pressure		
		Mean ± SD	Mean difference (95% CIs)	*P* 2-sided*	Mean ± SD	Mean difference (95% CIs)	*P* 2-sided*	Mean ± SD	Mean difference (95% CIs)	*P* 2-sided*
SPID (0–180)	Placebo	−15.3 ± 100.28	−657.7 (−714.83 to −600.52)	<.001	17.2 ± 127.33	−688.5 (−749.83 to −627.25)	<.001	23.8 ± 115.14	−664.78 (−725.32 to −604.25)	<.001
	TBF	642.4 ± 294.79			705.8 ± 308.93			688.5 ± 309.17		
AUC (0–180)	Placebo	−13.7 ± 87.68	−564.4 (−617.1 to −511.78)	<.001	15.7 ± 113.17	−590.2 (−646.19 to −534.25)	<.001	21.6 ± 102.49	−567.99 (−623.68 to −512.3)	<.001
	TBF	550.7 ± 273.1			606.0 ± 283.35			589.6 ± 285.61		
SPID (0–360)	Placebo	−34.7 ± 282.97	−1978.5 (−2073.99 to −1882.9)	<.001	36.4 ± 323.28	−2043.5 (−2150.92 to −1935.99)	<.001	50.1 ± 292.14	−2022.08 (−2122.86 to −1921.29)	<.001
	TBF	1943.8 ± 438.43			2079.8 ± 489.9			2072.2 ± 466.42		
AUC (0–360)	Placebo	−33 ± 266.65	−1860.13 (−1953.06 to −1767.2)	<.001	34.7 ± 305.58	−1920.95 (−2025.46 to −1816.45)	<.001	47.7 ± 276.37	−1900.18 (−1998.43 to −1801.92)	<.001
	TBF	1827.2 ± 431.74			1955.7 ± 481.99			1947.9 ± 459.79		

AUC = area under the curve; NRS = numerical pain rating scale; SPID = sum of pain intensity difference; TBF = turmeric-boswellia formulation.

* Mann–Witney test.

**Table 2:** Least square mean pain intensity difference of turmeric-boswellia formulation (TBF) from placebo for NRS (rest, movement and pressure).

Time	NRS rest										NRS movement										NRS pressure									
	TBF				Placebo				LSM Difference (P-T)	*P* *	TBF				Placebo				LSM Difference (P-T)	*P* *	TBF				Placebo				LSM Difference (P-T)	*P* *
	LSM	SE (LSM)	95% CI		LSM	SE (LSM)	95% CI				LSM	SE (LSM)	95% CI		LSM	SE (LSM)	95% CI				LSM	SE (LSM)	95% CI		LSM	SE (LSM)	95% CI			
			LL	UL			LL	UL					LL	UL			LL	UL					LL	UL			LL	UL		
0.5	7.3	0.11	6.9	7.6	7.5	0.11	7	7.8	0.2	>.05	7.7	0.08	8	7.9	8.2	0.08	8	7.9	0.5	.1557	7.7	0.08	8	7.9	8.2	0.08	8	8.4	0.5	.1557
1	5.9	0.11	5.6	6.2	7.8	0.11	8	8.1	1.9	.0037	6.2	0.08	6	6.4	8.1	0.08	6	6.4	1.9	.0008	6.2	0.08	6	6.4	8.1	0.08	8	8.3	1.9	.0008
1.5	4.8	0.11	4.5	5.1	7.7	0.11	7	8.1	2.9	.0007	5.1	0.08	5	5.3	8.2	0.08	5	5.3	3.2	.0001	5.1	0.08	5	5.3	8.2	0.08	8	8.5	3.2	.0001
2	3.7	0.11	3.4	4	7.8	0.11	8	8.1	4.1	.0002	3.6	0.08	3	3.8	8.2	0.08	3	3.8	4.6	<.001	3.6	0.08	3	3.8	8.2	0.08	8	8.4	4.6	<.001
2.5	2.7	0.11	2.4	3	7.8	0.11	7	8.1	5	.0001	2.7	0.08	2	2.9	8.2	0.08	2	2.9	5.5	<.001	2.7	0.08	2	2.9	8.2	0.08	8	8.4	5.5	<.001
3	1.9	0.11	1.5	2.2	7.8	0.11	8	8.1	5.9	<.001	1.7	0.08	2	2	8.1	0.08	2	2	6.4	<.001	1.7	0.08	2	2	8.1	0.08	8	8.3	6.4	<.001
3.5	1.4	0.11	1.1	1.7	7.8	0.11	7	8.1	6.3	<.001	1.3	0.08	1	1.6	8.2	0.08	1	1.6	6.8	<.001	1.3	0.08	1	1.6	8.2	0.08	8	8.4	6.8	<.001
4	1.1	0.11	0.8	1.4	7.8	0.11	8	8.1	6.7	<.001	1	0.08	1	1.2	8.1	0.08	1	1.2	7.1	<.001	1	0.08	1	1.2	8.1	0.08	8	8.3	7.1	<.001
4.5	0.8	0.11	0.5	1.1	7.8	0.11	7	8.1	6.9	<.001	0.9	0.08	1	1.1	8.1	0.08	1	1.1	7.2	<.001	0.9	0.08	1	1.1	8.1	0.08	8	8.4	7.2	<.001
5	0.5	0.11	0.2	0.8	7.8	0.11	7	8.1	7.2	<.001	0.6	0.08	0	0.8	8.1	0.08	0	0.8	7.5	<.001	0.6	0.08	0	0.8	8.1	0.08	8	8.3	7.5	<.001
5.5	0.3	0.11	0	0.6	7.8	0.11	8	8.1	7.5	<.001	0.5	0.08	0	0.7	8.2	0.08	0	0.7	7.7	<.001	0.5	0.08	0	0.7	8.2	0.08	8	8.4	7.7	<.001
6	0.2	0.11	-0	0.5	7.8	0.11	8	8.1	7.6	<.001	0.2	0.08	0	0.5	8.2	0.08	0	0.5	8	<.001	0.2	0.08	0	0.5	8.2	0.08	8	8.4	8	<.001

LSM = least square mean; NRS = numerical rating scale; P = placebo; T = treatment; TBF = turmeric-boswellia formulation.

* *P*-value, 2-sided Bonferroni.

**Table 3:** Response profile for pain improvement for NRS at Rest, movement and pressure.

Time	NRS Rest							NRS movement							NRS pressure						
	AUC		% improvement					AUC		% improvement					AUC		% improvement				
	Placebo	TBF	Placebo	TBF	Difference (P-T)	95% CI of difference		Placebo	TBF	Placebo	TBF	Difference (P-T)	95% CI of difference		Placebo	TBF	Placebo	TBF	Difference (P-T)	95% CI of difference	
						LCL	UCL						LCL	UCL						LCL	UCL
0.5	130.59	976.63	1.31	9.77	−8.46	−8.51	−8.41	248.36	965.75	2.48	9.66	−7.17	−7.23	−7.12	278.5	984.73	2.79	9.85	−7.06	−7.12	−7.01
1	118.68	2654.36	1.19	26.54	−25.36	−25.43	−25.28	313.98	2771.69	3.14	27.72	−24.58	−24.66	−24.49	351.23	2589.32	3.51	25.89	−22.38	−22.46	−22.3
1.5	127.49	4322.7	1.27	43.23	−41.95	−42.03	−41.88	308.9	4314.95	3.09	43.15	−40.06	−40.14	−39.98	349.92	4240.81	3.5	42.41	−38.91	−38.99	−38.83
2	115.08	5776.29	1.15	57.76	−56.61	−56.67	−56.55	314.86	6006.59	3.15	60.07	−56.92	−56.98	−56.85	342.17	5777.81	3.42	57.78	−54.36	−54.42	−54.29
2.5	162.98	6928.88	1.63	69.29	−67.66	−67.72	−67.6	350.94	7112.08	3.51	71.12	−67.61	−67.67	−67.55	382.67	6957.63	3.83	69.58	−65.75	−65.81	−65.69
3	159.76	7912.62	1.6	79.13	−77.53	−77.58	−77.48	347.32	8016.38	3.47	80.16	−76.69	−76.74	−76.64	381.41	7944.06	3.81	79.44	−75.63	−75.68	−75.58
3.5	188.7	8406.32	1.89	84.06	−82.18	−82.22	−82.13	365.78	8525.84	3.66	85.26	−81.6	−81.64	−81.56	407.86	8431.39	4.08	84.31	−80.24	−80.28	−80.19
4	190.21	8766.48	1.9	87.66	−85.76	−85.8	−85.72	366.36	8790.92	3.66	87.91	−84.25	−84.28	−84.21	409.35	8771.57	4.09	87.72	−83.62	−83.66	−83.58
4.5	207.87	9031.2	2.08	90.31	−88.23	−88.26	−88.2	382.65	8955.95	3.83	89.56	−85.73	−85.77	−85.7	416.98	9003.77	4.17	90.04	−85.87	−85.9	−85.83
5	249.02	9393.06	2.49	93.93	−91.44	−91.47	−91.41	427.34	9280.84	4.27	92.81	−88.54	−88.57	−88.5	455.56	9396.5	4.56	93.97	−89.41	−89.44	−89.38
5.5	246.83	9634.37	2.47	96.34	−93.88	−93.9	−93.85	425.23	9533.1	4.25	95.33	−91.08	−91.11	−91.05	454.33	9636.1	4.54	96.36	−91.82	−91.85	−91.79
6	245.82	9785.16	2.46	97.85	−95.39	−95.42	−95.37	426.56	9778.6	4.27	97.79	−93.52	−93.55	−93.49	456.02	9784.14	4.56	97.84	−93.28	−93.31	−93.25

NRS = numerical rating scale; P = placebo; T = treatment; TBF = turmeric-boswellia formulation.

### 3.2. Onset of analgesia

**Figure 2.**
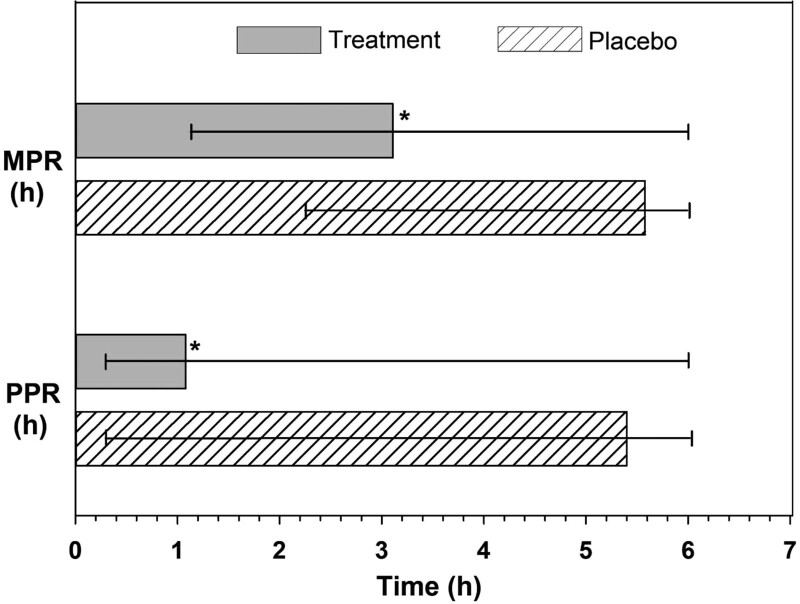
Comparison of mean time to achieve perceptible pain relief (PPR) and meaningful pain relief (MPR) in treatment turmeric-boswellia formulation (TBF) and placebo groups.

The onset of analgesia was fast and highly significant in the TBF group with 99.1% of participants experiencing a PPR and 95.7% of participants achieving MPR compared to 10.4% of participants experiencing PPR and 1.7% achieving MPR in the placebo group (*P* < .0001). As the pain relief was relatively low in the placebo group, the median pain relief time could not be estimated from the observed data. RMST analysis showed a PPR of 68.5 minutes and MPR of 191.6 minutes in TBF group and 340.7 and 358.1 minutes in the placebo group, respectively (Fig. 2). The RMST ratio indicates that a person in TBF group experienced a PPR of 4.98 times faster and achieved an MPR of 1.87 times faster than the placebo. The RMLT ratio of 0.07 (PPR) and 0.01 (MPR) showed that the placebo group experienced 93% and 99% less pain-free time, respectively. A low RMST or a high RMLT in MPR and PPR indicated a lesser duration of pain in the TBF group and showed an overall benefit compared to the placebo group (Table 4). Graphical representation of individuals achieving PPR and MPR is given in (Figs. S4 and S5, Supplementary Digital Content 4, http://links.lww.com/MD/H115).

**Table 4:** Median survival time, restricted mean survival time, and restricted mean lost time analysis of onset of analgesia using the double-stopwatch method.

Pain relief (event) time	Perceptible pain relief		Meaningful pain relief		
	TBF	Placebo	TBF		Placebo
Symptom resolved (n = 116)	115	12	111		2
Median survival time	60	NE	180		NE
Restricted mean survival time (RMST ± SE)	68.5 ± 4.562	340.7 ± 6.076	191.6 ± 7.581		358.1 ± 1.804
95% CL (LL–UL)	59.5–77.4	328.8–352.6	176.7–206.4		354.6–361.6
RMST difference ± SE (placebo-TBF) (*P*-value)	272.3 ± 7.598 (*P* < .0001)		166.6 ± 7.793 (*P* < .0001)		
95% CL (LL–UL)	257.4–287.1		151.3–181.8		
RMST ratio ± SE (placebo/TBF)	4.98 ± 0.069 (*P* < .0001)		1.87 ± 0.04 (*P* < .0001)		
95% CL (LL–UL)	4.35–5.7		1.73–2.02		
Restricted mean lost time (RMLT ± SE)	291.5 ± 4.562	19.3 ± 6.076	168.4 ± 7.581		1.9 ± 1.804
95% CL (LL–UL)	282.6–300.5	7.4–31.2	153.6–183.3		−1.6 to 5.4
RMLT ratio (placebo/TBF) ± SE	0.07 ± 0.315 (*P* < .0001)		0.01 ± 0.952 (*P* < .0001)		
95% CL (LL–UL)	0.04–0.12		0–0.07		

Total available time = 360 min.

NE = not estimable; RMLT = restricted mean survival time lost; RMST = restricted mean survival time; TBF = turmeric-boswellia formulation.

**Figure 3.**
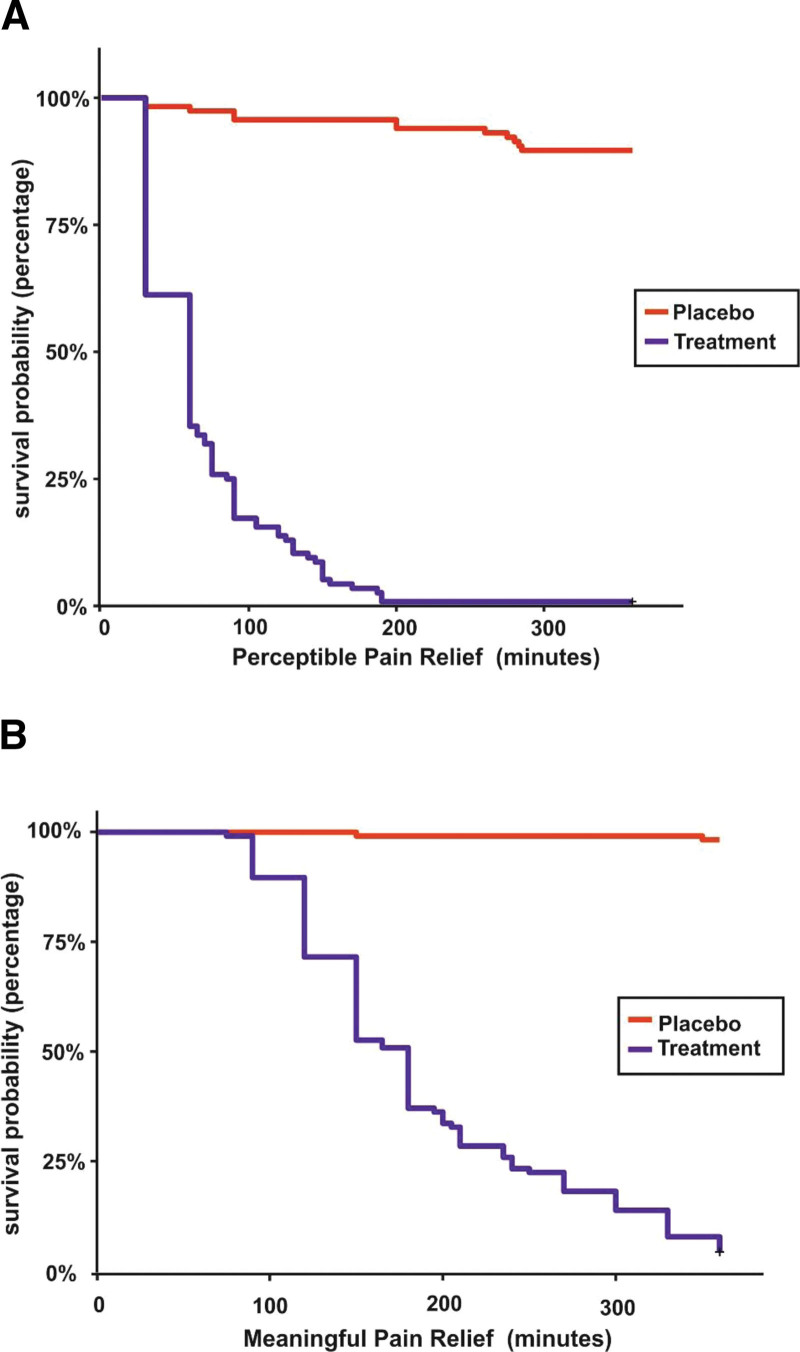
Comparison of treatment turmeric-boswellia formulation (TBF) and placebo group using Kaplan-Meier survival plot of onset of analgesia. (A) Perceptible pain relief. (B) Meaningful pain relief.

From the KM survival plots for PPR, 45 (39%) participants in the TBF group experienced PPR as early as 30 minutes and 115 (99%) within 190 minutes, whereas in the placebo group, only 2 (1.7%) participants experienced PPR as early as 30 minutes and 12 (10.3%) within 285 minutes. The earliest MPR was reached within 75 minutes (1 subject) and 111 (96%) participants had MPR within 360 minutes while the placebo had only 2 events (1.7%) within 360 minutes (Fig. 3) (Tables S3–S6, Supplementary Digital Content 5, http://links.lww.com/MD/H116).

The Log-rank hazard ratio for PPR and MPR indicated a significant difference (*P* < .001) in symptom resolution between the groups. Cox regression analysis estimated PPR and MPR with NRS rest as a covariate and showed a significant difference (*P* < .001) in symptom resolution indicating that a person receiving TBF was 83 and 272 times more likely to experience PPR and MPR compared with placebo whereas the corresponding figures for PPR and MPR in log-rank test was 19 and 107 times (Tables S7–S9, Supplementary Digital Content 6, http://links.lww.com/MD/H117). This strongly suggests that a difference between the groups exists which is clinically important. The proportional hazard assumption was checked graphically using Schoenfeld residual test and represented in (Figs. S6 and S7, Supplementary Digital Content 7, http://links.lww.com/MD/H118).

### 3.3. Pain relief scale

TOTPAR6_rest, move, pres_ showed a significant difference (*P* < .0001) between the 2 groups demonstrating better efficacy for TBF in obtaining pain relief. The effect of treatment in terms of NNT in TBF group for 50% of maximum pain relief was 1.1 in rest, movement, and pressure. The number of participants with ≥ 50% of maximum TOTPAR was 108, 109, and 109 in the TBF group compared with 3, 1, and 1 in the placebo (*P* < .001 for PRS_rest, move,pres_) (Table 5).

**Table 5:** Comparison of total pain relief between turmeric-boswellia formulation (TBF) and placebo and number needed to treat.

Scale	Parameter	TBF (n)	Placebo (n)	*P* *	TBF proportion (n/N)	Placebo proportion (n/N)	NNT	*P* 2-sided^†^
PRS_rest_	Median TOTPAR(95% CI)	18 (17–19)	0 (0–0)	<.00001				
	≥50% of Max TOTPAR	108	3		0.9310	0.0259	1.1048	<.00001
	<50% of Max TOTPAR	8	113		0.0689	0.9741		
PRS_move_	Median TOTPAR(95% CI)	18.25 (17–19)	0 (0–0)	<.00001				
	≥50% of Max TOTPAR	109	1		0.9397	0.0086	1.0741	<.00001
	<50% of Max TOTPAR	7	115		0.0603	0.9914		
PRS_pres_	Median TOTPAR	18 (17–19)	0 (0–0)	<.00001				
	≥50% of Max TOTPAR	109	1		0.9397	0.0086	1.07407	<.00001
	<50% of Max TOTPAR	7	115		0.0603	0.9914		

NNT = number needed to treat; PRS = pain relief scale; TBF = turmeric-boswellia formulation; TOTPAR = total pain relief.

* Mann–Whitney *U* or Wilcoxon rank-sum test between groups.

† Wald Chi-square test.

The RMST to achieve maximum pain relief by KM analysis was 194, 197.7, and 194.2 minutes, respectively, for rest, movement, and pressure for TBF compared with 345.5, 345.5, and 356.7, respectively, for placebo which was statistically significant (*P* < .0001). A high RMST or low RMLT in PRS_rest,move,pres_ indicates faster pain relief in the TBF group and shows overall benefit compared with the placebo group (Table 6). In the TBF group, 50% of participants experienced maximum pain relief of 180, 182, and 180 minutes (median time) for rest, movement, and pressure, respectively. The median time was not estimable in the placebo group.

**Table 6:** Restricted mean survival time to achieve maximum pain relief and restricted mean time lost for pain-free time from the categorical pain relief scale (PRS).

Scale	Group	Subjects (n)		Τime (min)	RMST ± SE (95% CI) (min)	RMTL ± SE (95% CI) (min)	Between-group mean survival comparison			
		Symptom resolved	Total				RMST difference (95% CI) (min) (P-TBF)	RMST ratio (95% CI) (P/TBF)	RMTL ratio (95% CI) (P/TBF)	*P*
PRS on rest	TBF	108	116	360	194 ± 7.40 (179.5–208.5)	166 ± 7.40 (151.5–180.5)	151.5 ± 8.94 (134–169)	1.78 ± 0.04(1.64–1.93)	0.09 ± 0.35(0.04–0.17)	<.0001*
										<.0001^†^
	Placebo	11	116	360	345.5 ± 5.01 (335.7–355.3)	14.5 ± 5.01 (4.7–24.3)				<.0001^‡^
PRS on movement	TBF	114	116	360	197.7 ± 7.40 (183.2–212.2)	162.3 ± 7.40 (147.8–176.8)	155.5 ± 8.03(139.8–171.2)	1.79 ± 0.04 (1.66–1.93)	0.04 ± 0.46 (0.02–0.1)	<.0001*
										<.0001^†^
	Placebo	9	116	360	353.2 ± 3.10(347.1–359.3)	6.8 ± 3.10 (0.7–12.9)				<.0001^‡^
PRS on pressure	TBF	110	116	363	194.2 ± 7.58 (179.3–209)	168.8 ± 7.58 (154–183.7)	162.6 ± 8.18 (146.5–178.6)	1.84 ± 0.04 (1.7–1.99)	0.04 ± 0.49 (0.01–0.1)	<.0001*
										<.0001^†^
	Placebo	8	116	363	356.7 ± 3.08 (350.7–362.8)	6.3 ± 3.08 (0.2–12.3)				<.0001^‡^

P = placebo; RMST = restricted mean survival time to maximum pain relief; RMTL = restricted mean time lost to maximum pain relief; TBF = turmeric-boswellia formulation.

* Between-group *P*-value of RMST difference.

† Between-group *P*-value of RMST ratio.

‡ Between-group *P*-value of RMTL ratio.

The repeated measure proportional odds logistic regression analysis showed an increase in pain relief with time represented by a negative time coefficient, which was significant in both TBF (*P* < .0001) and placebo (*P* < .05). A significant placebo effect can be seen with this analysis but the odds of experiencing complete pain relief in the same time period are negligible compared to TBF. The cumulative probability odds of getting complete pain relief with placebo compared with TBF were 0.00025, 0.00025, and 0.00024 times for rest, movement, and pressure, respectively (Table 7).

**Table 7:** Numbers of subjects according to the category of pain relief with cumulative probability odds ratio and repeated proportional odds (0–6 h) from pain relief scale (PRS).

Scale	Group	Numbers of subjects according to the category of pain relief					Cumulative probability odds ratio*			Repeated proportional odds (0–6 h)^†^	
		None	Little	Some	A Lot	Complete		Odds ratio for complete pain relief(P/T)	Odds ratio for no pain rerelief(P/T)	Coefficient	*P*
PRS at rest	TBF	1	0	0	7	108		0.00025	3966.142	−0.5856	<.0001
	Placebo	105	10	0	0	1				−0.1743	.0304
PRS at movement	TBF	1	0	0	9	106		0.00025	3947.726	−0.5812	<.0001
	Placebo	107	8	0	0	1				−0.2253	.0429
PRS at pressure	TBF	1	0	0	7	108		0.00024	4093.149	−0.5963	<.0001
	Placebo	108	7	0	0	1				−0.2253	.0429

PRS = pain relief scale; TBF = turmeric-boswellia formulation

* Between-group analysis odds ratio.

† Within-group analysis.

### 3.4. Short form of McGill pain questionnaire

TBF showed a statistically significant reduction in total, sensory and affective domains of MPQ (*P* < .0001), VAS (*P* < .0001), PPI (*P* < .0001) comparing posttreatment with pretreatment while placebo had no significant change in MPQ (*P* > .05), whereas a significant change was observed in VAS (*P* = .001) and PPI (*P* = .005). Standardized response mean value >0.80 shows a clinically significant improvement and the TBF group had a value >0.8 in MPQ, VAS, and PPI, whereas the placebo group had <0.5 in MPQ, VAS, and PPI indicating no beneficial change. A change of 13.7 mm in VAS shows a minimum clinically important difference (MCID) and TBF showed a reduction greater than MCID (mean difference 78.6 mm), whereas the change in placebo was less than MCID (Table 8).

**Table 8:** McGill short-form questionnaire comparison between turmeric-boswellia formulation (TBF) and placebo group.

MPQ Score	TBF					Placebo				
	Mean ± SD	Mean difference ± SD	95 % CL (LL–UL)	*P* *	SRM^†^	Mean ± SD	Mean difference ± SD	95 % CL (LL–UL)	*P* *	SRM^†^
MPQ sensory (scale 0–33)										
Pretreatment	11.1 ± 6.08	10.9 ± 6.17	9.8–12	<.001	1.77	11.2 ± 5.8	−0.2 ± 1.08	−0.4 to 0	.0857	0.19
Posttreatment	0.2 ± 0.95					11.4 ± 5.98				
MPQ affective (scale 0–12)										
Pretreatment	3.1 ± 2.62	3 ± 2.61	2.5–3.5	<.001	1.15	3 ± 2.54	0 ± 1.32	−0.2 to 0.3	.3775	0
Posttreatment	0.1 ± 0.58					3 ± 2.45				
Total MPQ (scale 0–45)										
Pretreatment	14.2 ± 5.83	13.9 ± 6.01	12.8–15	<.001	2.31	14.2 ± 5.37	−0.1 ± 1.98	−0.5 to 0.2	.1429	0.1
Posttreatment	0.3 ± 1.46					14.4 ± 5.66				
VAS										
Pretreatment	80.7 ± 12.46	78.6 ± 14.27	75.9–81.2	<.001	5.51	80.5 ± 12.06	−1 ± 9.86	−2.8 to 0.8	.0011	0.1
Posttreatment	2.1 ± 8.5					81.5 ± 14.84				
PPI										
Pretreatment	4.3 ± 0.69	4.1 ± 0.82	3.9–4.2	<.001	4.88	4.1 ± 0.76	−0.1 ± 0.53	−0.2 to 0	.0052	0.19
Posttreatment	0.2 ± 0.5					4.2 ± 0.91				

MPQ = McGill pain questionnaire; SD = standard deviation; SRM = standardized response mean; TBF = turmeric-boswellia formulation.

* Wilcoxon signed-rank test.

† SRM = mean change/SD of the change.

The measurement error in MPQ was estimated by SDD in each domain. In the TBF group, out of 116 participants, 61 participants had an MPQ total score more than SDD68 and 58 participants improved, whereas, in the placebo group, 116 participants had the chance of improving MPQ total score more than SDD68 and only 3 participants improved. S_w_% calculated as a percentage of the total score of each domain was >20 for all domains in the TBF group indicating that the improvement observed is due to treatment effect and not due to measurement error. The higher ICC (3,1) values compared with ICC (1,1) in the TBF group showed a difference between pre- and postscores in MPQ indicating a reliable reduction in pain but in the placebo group, the ICC values indicated no change between pre- and postscores. Higher S_w_% in the TBF group indicated that sensory domain scores were reduced more than other domains of MPQ, whereas, in the placebo group, more reduction was observed in affective domain scores (Table 9).

**Table 9:** Test-retest reliability of MPQ scores in subjects with musculoskeletal pain under placebo and turmeric-boswellia formulation (TBF) treatment group.

MPQ score	ICC (1,1) (95% CI)	ICC (2,1) (95% CI)	ICC (3,1) (95% CI)	S_w_	S_w_%*	SDD_68_	Improved beyond SDD	Improvable beyond SDD
							n (%)	N (%)
TBF								
Sensory (0–33)	−0.61 (−0.7141 to −0.4846)	0 (−0.03992 to 0.04987)	0 (−0.1864 to 0.1768)	8.85	27	12.5	61 (53)	63 (54)
Affective (0–12)	−0.35 (−0.5035 to −0.1853)	0.02 (−0.05766 to 0.1188)	0.05 (−0.1322 to 0.2302)	2.82	23	4	11 (9)	12 (10)
Total (0–45)	−0.73 (−0.8035 to −0.6303)	0 (−0.02484 to 0.03366)	0 (−0.1820 to 0.1812)	10.72	24	15.1	58 (50)	61 (53)
Placebo								
Sensory (0–33)	0.98 (0.9752–0.9880)	0.98 (0.9751–0.9880)	0.98 (0.9756–0.9882)	0.77	2	1.1	3 (3)	104 (90)
Affective (0–12)	0.86 (0.8063–0.9020)	0.86 (0.8061 to 0.9021)	0.86 (0.8048 to 0.9014)	0.93	8	1.3	2 (2)	86 (74)
Total (0–45)	0.94 (0.9082–0.9548)	0.94 (0.9082–0.9548)	0.94 (0.9079–0.9547)	1.4	3	2	3 (3)	116 (100)

SDD_68_ = 1.41 × S_w_.

S_w_% = S_w_ divided by total score of each domain × 100.

ICC = intraclass correlation coefficient; N = number of subjects improvable beyond SDD; n = number of subjects improved beyond SDD; SDD_68_ = smallest detectable difference at 68% confidence interval; S_w_ = within-subject standard deviation.

* Chance of measurement error: ≤5% ‘‘very good”, >5% and ≤10% ‘‘good”, >10% and ≤20% ‘‘doubtful”, and >20% ‘‘negative”.

## 4. Discussion

The study selection criteria restricted the entry if the participant had a pain score of <5 on a 0 to 10 pain scale. Eighty percent of the study participants recorded a pain 7 to 9 on the NRS scale taken (83% [97 out of 116] in the TBF group and 77% [90 out of 116] in the placebo group). TBF is a formulation composed of turmeric and boswellia. Curcumin inhibits cyclooxygenase 2 and prostaglandin E2 in a concentration and time-dependent manner. In vivo studies showed that curcumin produced an analgesic effect via antagonism of transient receptor potential vanilloid 1, which plays an important role in nociception.^[26]^ A systematic review and meta-analysis of 8 RCT’s found a significant reduction in pain by curcuminoids (*P* = .04).^[27]^ Analgesic activity has also been previously reported for boswellia and boswellic acids, including AKBA.^[28]^ In the present study, the analgesic effect of TBF may be due to the presence of multiple natural anti-inflammatory ingredients, which act to improve acute and neuroinflammation, in addition to modulating multiple pain pathways. The fast onset of analgesia (PPR 68.5 minutes and MPR 191.6 minutes) observed with TBF in our study may be explained in this context.

Pain and emotions interact in several ways that influence cognitive appraisals of the pain state.^[29,30]^ TBF exerted a considerable positive effect on both affective and sensory domains of pain. The present study data also provide the relative magnitudes of sensory-intensive and affective dimensions of different types of clinical pain before and after the treatment with TBF versus placebo. The higher ICC values for MPQ total and sensory domain compared with the affective domain in the placebo group suggests a placebo effect, which conforms with findings from other studies.^[31–33]^ The usual contention of error of measurement with subjective pain questionnaires was overcome by analyzing treatment change beyond measurement error by way of SDD_68_. In the placebo group, error of measurement was found to be within acceptable limits as defined by Ostelo et al^[34]^ for the total, sensory and affective scales as S_w_ values, related to the total score of the corresponding scale, were ≤10%. In the TBF group, the error of measurement was >20% due to high variability between the 2 measurements, indicating the effectiveness of TBF in reducing pain experienced by the participants.

The repeated proportional odds logistic regression used showed a continuous improvement in pain relief, which was highly significant with 93% of participants having ≥ 50% of maximum TOTPAR6. The NNT is a valuable measure that describes the number of participants to be treated with an analgesic intervention to have at least 50% pain relief over 6 hours. The best NNT would, of course, be 1 but generally, NNTs between 2 and 5 are indicative of effective analgesic treatments.^[18]^ In our study, the NNT was 1.1 for PRS at rest, movement, and pressure, which indicates an excellent analgesic effect.

The possibility of a placebo effect in the present study cannot be disregarded as the pain relief categorical scale had 9.5% with pain symptom resolution at rest, 7.8% on movement, and 6.9% on the pressure in the placebo group (RMST analysis table). Previous studies on acute pain and exercise-related studies frequently report a minimal placebo effect, often <5%. Controversy exists regarding whether placebos alter sensory pain transmission, pain effect, or simply produce compliance with the suggestions of investigators.^[35]^ Placebos are more effective when the patients have high expectations of treatment or are under stress and anxiety related to their pain.

Pain relief category scales have been reported as more sensitive to small reductions in pain compared with the pain intensity category scales.^[36]^ Categorical PRSs rely on the patient’s memory of pain at the baseline period. Emotions exert strong assimilative effects on memory^[37]^ and could, therefore, be important in the processing of pain memory. Hence, the placebo effect may be more pronounced in the PRS versus other scales. In our study, both TBF and placebo provided relief in pain indicated by statistically significant negative time coefficients in repeated measure proportional odds logistic regression but placebo had lower odds of experiencing complete pain relief (0.00025 times that of TBF). TBF alleviates exercise-induced acute musculoskeletal pain as measured by NRS, which showed a fast onset of analgesia. It gives relief in pain intensity as well as improves psychological wellness as measured by MPQ.

Several new approaches to pain treatment revolve around the use of mechanisms to destroy or exhaust neurons involved in pain transmission. One approach is to use targeted neurotoxins to cause neuronal death. Another approach uses the TRPV1 to target neurons involved in pain. The roots of turmeric contain active constituents called curcuminoids, mainly curcumin. The exact mechanism for reducing pain is unknown; however, it is thought that curcumin can inhibit transient receptor potential vanilloid (TRPV1)-mediated pain.^[38]^ When drugs that bind to TRPV1 help in calcium influx that results in an inability of the neuron to function. If the activation of TRPV1 occurs for long enough or is intense enough, the resulting calcium influx can cause the neurons to degenerate and undergo apoptosis. Boswellic acids appeared to exert a specific in vitro inhibitory activity on 5-lipoxygenase, with little effect on cyclooxygenase (which produces prostaglandins) or 12-lipoxygenase.^[39]^ The inhibitory effect of AKBA is reversible, and increased levels of arachidonic acid as a substrate for COX-1 impair the efficacy.^[39]^ Boswellic acids inhibit the transformation of arachidonic acid to leukotrienes via 5-lipoxygenase but can also enhance the liberation of arachidonic acid in human leukocytes and platelets.^[40]^ The mechanism of action is, therefore, quite distinct from conventional treatments, which inhibit prostaglandin production.

### 4.1. Clinical significance

Traditionally, clinicians have relied heavily on the use of non-steroidal anti-inflammatory drugs to treat the pain as numerous studies have proven these agents are effective. Of the newer agents, some of the COX-2 inhibitors are withdrawn from the market over concerns of its cardiovascular side effects,^[41]^ casting a large cloud over the future of this class of drugs.^[42]^ Adverse effects also appear to plague the use of other recent additions like calcium channel blocker, which is poorly tolerated by some patients due to its central nervous system adverse effects, especially somnolence and dizziness.^[43]^ The investigational product used in the study is an option for the healthcare providers who opt for complementary therapy and researchers working on novel pain relief products.

### 4.2. Strengths and limitations of the study

CPRA was analyzed with minimum percentage improvement in pain from baseline with 95% CI calculated using bootstrapping with 5000 iterations helped to generalize the results of the study. Stratification at a 1% increment on the response criteria (pain reduction in NRS) helps to determine the responders at any specific response rate for further analysis and research. This study depends on the subjective data from the participants with no objective measures like biomarker analysis and the lack of positive control is the limitation of the study. Further research can be planned on a study population from a worldwide community.

## 5. Conclusion

Exercise-induced acute musculoskeletal pain of moderate-to-severe intensity can be effectively relieved by single dose of 1000 mg of TBF (Rhuleave-K) in about 3 hours signifying its strong analgesic activity. The onset of pain relief was fast and highly significant in the TBF group having a mean PPR at 68.5 minutes and MPR at 191.6 minutes compared with the placebo group. TBF can be suggested as an effective and safe natural alternative for the management of acute pain.

## Acknowledgments

The authors would like to acknowledge Arjuna Natural Pvt Ltd., Kerala, India, for providing the test product (Rhuleave-K) and placebo capsules.

## Author contributions

GHR was involved in study design, investigation, and analysis of the study, and wrote the original draft of the manuscript. MM and SS were involved in investigation, project administration and reviewed the manuscript. SKK, AG and IB contributed to study design, planning, investigation and reviewed the manuscript. All authors read and approved the final manuscript.

## Supplementary Material


