# Supplementary material for: Fast pain relief in exercise-induced acute musculoskeletal pain by turmeric-boswellia formulation: A randomized placebo-controlled double-blinded multicentre study
Source: Medicine (Baltimore). 2022 Sep 2;101(35):e30144. doi: 10.1097/MD.0000000000030144 (PMC9439841; doi:10.1097/MD.0000000000030144)
Supplement: Supplementary file 1 [file medi-101-e30144-s001.pdf]

Supplementary table S1. Time points and schedule of activities				
Activities	Screening (Pre-dose)	Randomization 0 h	Post dose 0 – 6 h (every 30 min)	After 6 h
Informed consent	**X**			
Demographics/ Medical History	**X**			
Physical examination/ Vitals	**X**			
NRS (at rest)	**X**		**X**	
NRS (on movement)	**X**		**X**	
NRS (with pressure)	**X**		**X**	
McGill-SFQ	**X**			**X**
IP allocation		**X**		
PRS (at rest)			**X**	
PRS (on movement)			**X**	
PRS (with pressure)			**X**	
Onset of Analgesia			**X**	

NRS = Numerical Rating Scale
PRS = Pain Relief Scale
IP = Investigational Product
SFQ= Short Form Questionnaire
