# Supplementary material for: Fast pain relief in exercise-induced acute musculoskeletal pain by turmeric-boswellia formulation: A randomized placebo-controlled double-blinded multicentre study
Source: Medicine (Baltimore). 2022 Sep 2;101(35):e30144. doi: 10.1097/MD.0000000000030144 (PMC9439841; doi:10.1097/MD.0000000000030144)
Supplement: Supplementary file 2 [file medi-101-e30144-s002.pdf]

Supplementary table S2. Demographics and baseline data of Turmeric-Boswellia formulation (TBF) and placebo groups

	TBF (n=116)	Placebo (n=116)	#P value (2t)
	Mean ± SE	Mean ± SE	
Age	37.18 ± 1.19	37.55 ± 1.25	0.919
Height	166.85 ± 0.87	166.69 ± 0.81	0.747
Weight	69.81 ± 1.07	69.75 ± 1.08	0.846
NRS Rest	7.97 ± 0.11	7.66 ± 0.11	0.053*
NRS Movement	8.41 ± 0.10	8.28 ± 0.10	0.357*
NRS Pressure	8.44 ± 0.10	8.29 ± 0.09	0.266*

# Mann-Whitney U test.

* Kolmogorov-Smirnov Test for Comparing Distributions
