# Supplementary material for: Fast pain relief in exercise-induced acute musculoskeletal pain by turmeric-boswellia formulation: A randomized placebo-controlled double-blinded multicentre study
Source: Medicine (Baltimore). 2022 Sep 2;101(35):e30144. doi: 10.1097/MD.0000000000030144 (PMC9439841; doi:10.1097/MD.0000000000030144)
Supplement: Supplementary file 3 [file medi-101-e30144-s003.pdf]

Supplementary figure S1. Cumulative proportion of responder analysis - NRS at Rest

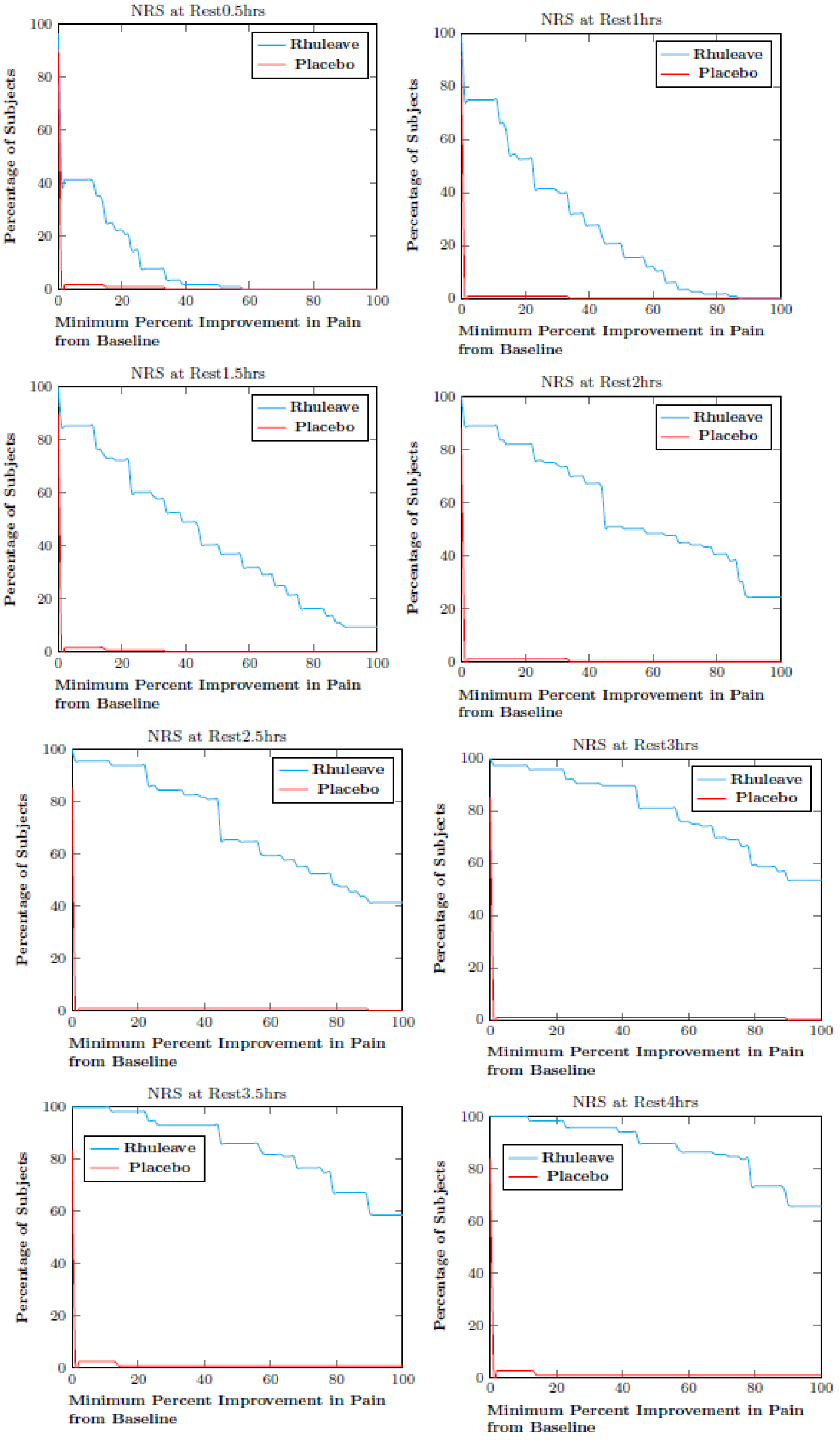

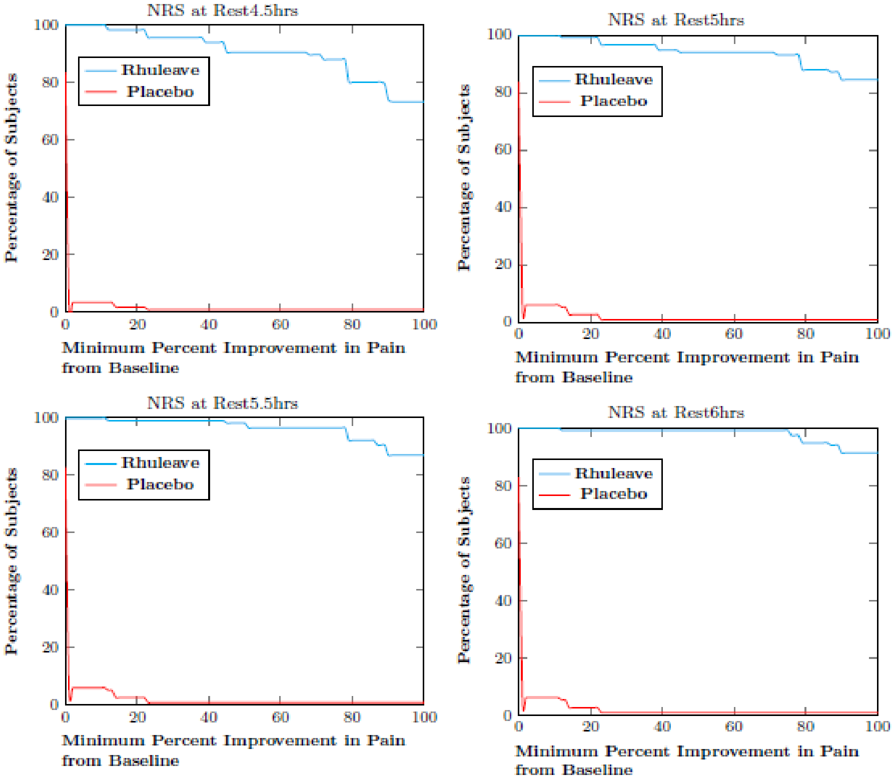
NRS at Rest4.5hrs
Rhuleave
Placebo
Percentage of Subjects
Minimum Percent Improvement in Pain from Baseline
NRS at Rest5hrs
Rhuleave
Placebo
Percentage of Subjects
Minimum Percent Improvement in Pain from Baseline
NRS at Rest5.5hrs
Rhuleave
Placebo
Percentage of Subjects
Minimum Percent Improvement in Pain from Baseline
NRS at Rest6hrs
Rhuleave
Placebo
Percentage of Subjects
Minimum Percent Improvement in Pain from Baseline

Supplementary figure S2. Cumulative proportion of responder analysis - NRS at Movement

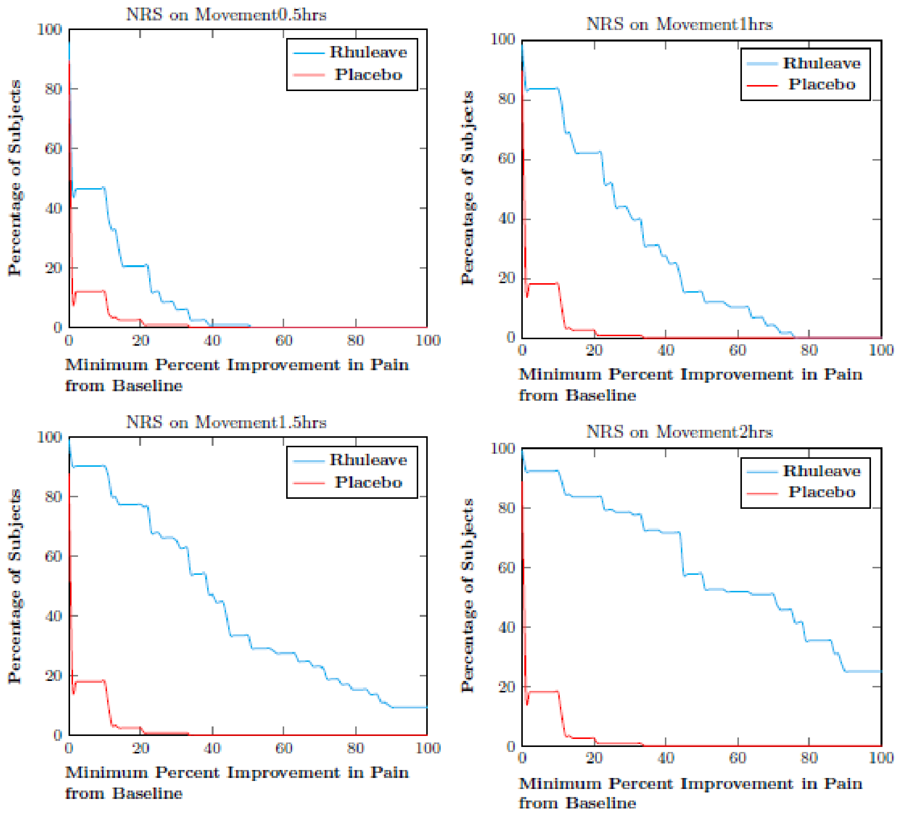

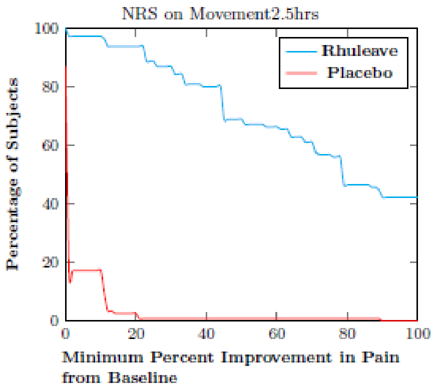
NRS on Movement2.5hrs
Rhuleave
Placebo
Percentage of Subjects
Minimum Percent Improvement in Pain from Baseline

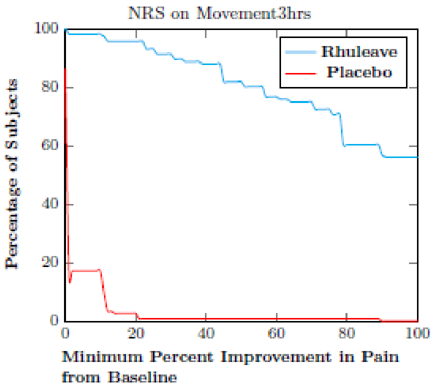
NRS on Movement3hrs
Rhuleave
Placebo
Percentage of Subjects
Minimum Percent Improvement in Pain from Baseline

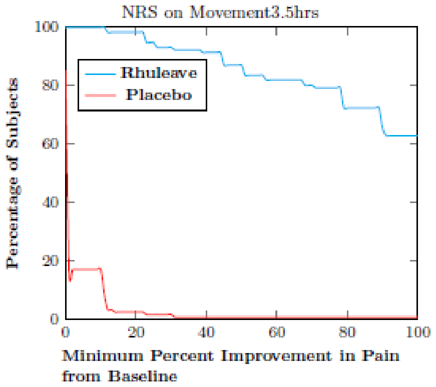
NRS on Movement3.5hrs
Rhuleave
Placebo
Percentage of Subjects
Minimum Percent Improvement in Pain from Baseline

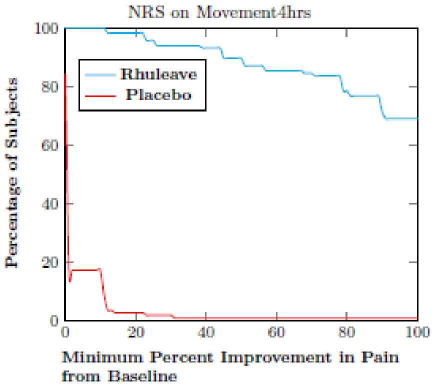
NRS on Movement4hrs
Rhuleave
Placebo
Percentage of Subjects
Minimum Percent Improvement in Pain from Baseline

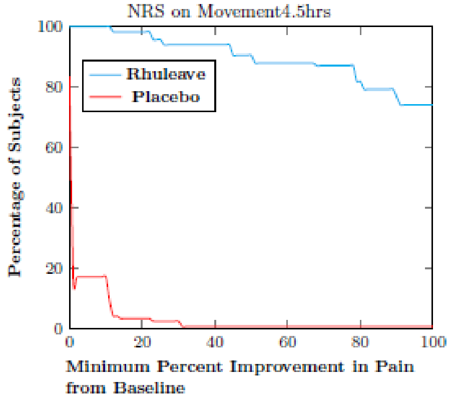
NRS on Movement4.5hrs
Rhuleave
Placebo
Percentage of Subjects
Minimum Percent Improvement in Pain from Baseline

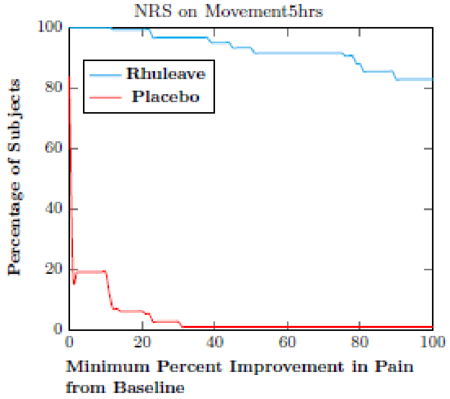
NRS on Movement5hrs
Rhuleave
Placebo
Percentage of Subjects
Minimum Percent Improvement in Pain from Baseline

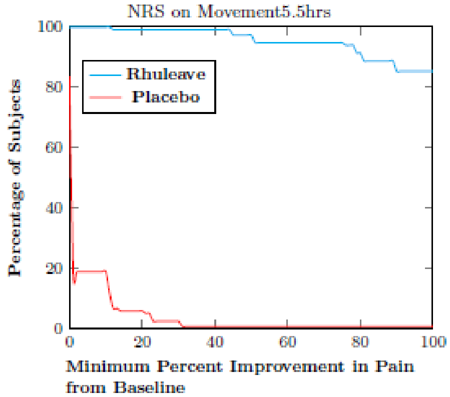
NRS on Movement5.5hrs
Rhuleave
Placebo
Percentage of Subjects
Minimum Percent Improvement in Pain from Baseline

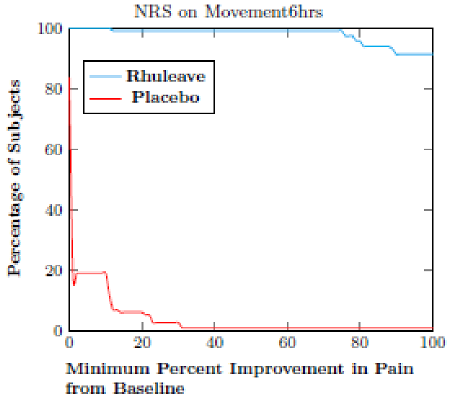
NRS on Movement6hrs
Rhuleave
Placebo
Percentage of Subjects
Minimum Percent Improvement in Pain from Baseline

Supplementary figure S3. Cumulative proportion of responder analysis - NRS at Pressure

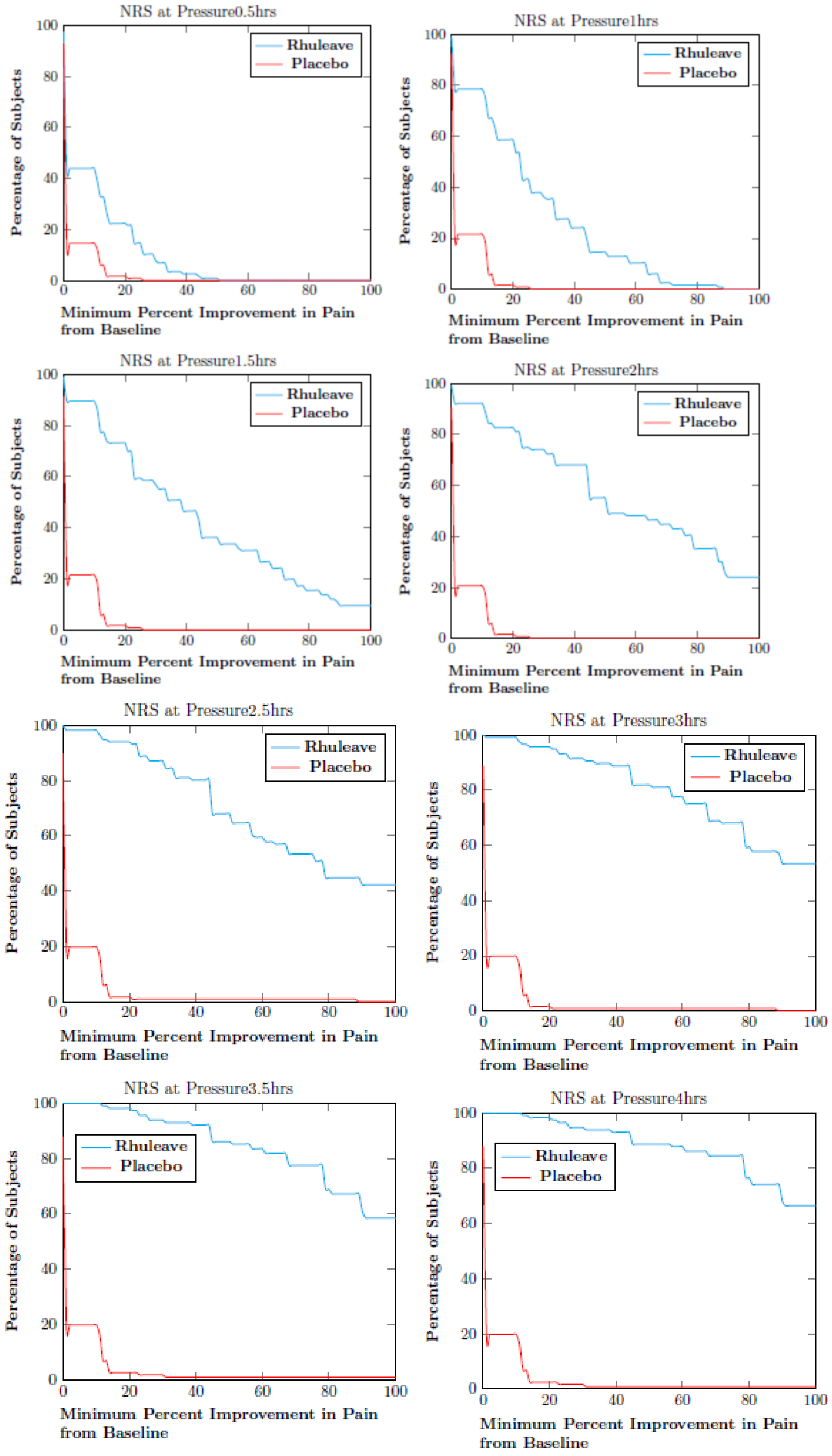

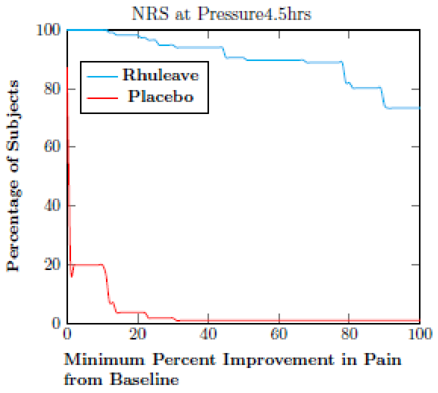
NRS at Pressure4.5hrs
Rhuleave
Placebo
Percentage of Subjects
0
20
40
60
80
100
Minimum Percent Improvement in Pain from Baseline

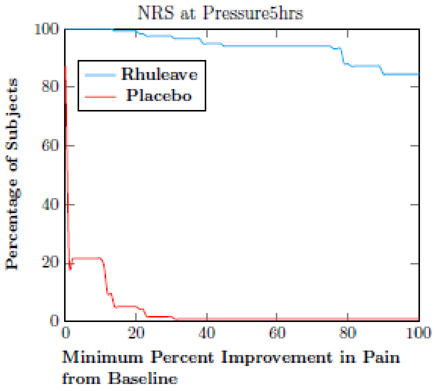
NRS at Pressure5hrs
Rhuleave
Placebo
Percentage of Subjects
0
20
40
60
80
100
Minimum Percent Improvement in Pain from Baseline

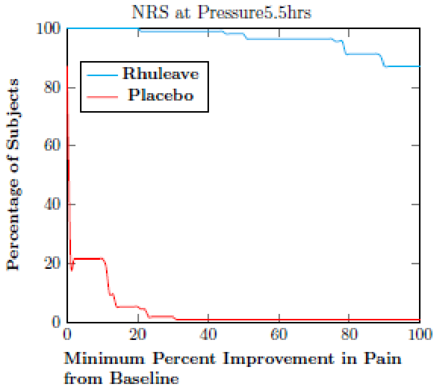
NRS at Pressure5.5hrs
Rhuleave
Placebo
Percentage of Subjects
0
20
40
60
80
100
Minimum Percent Improvement in Pain from Baseline

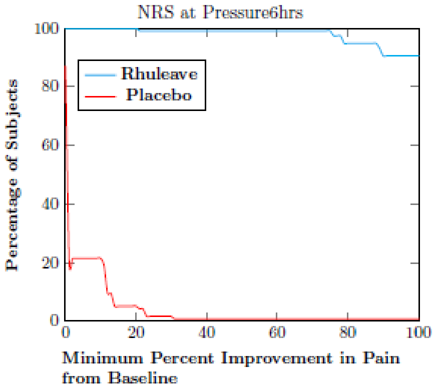
NRS at Pressure6hrs
Rhuleave
Placebo
Percentage of Subjects
0
20
40
60
80
100
Minimum Percent Improvement in Pain from Baseline
