# Supplementary material for: Fast pain relief in exercise-induced acute musculoskeletal pain by turmeric-boswellia formulation: A randomized placebo-controlled double-blinded multicentre study
Source: Medicine (Baltimore). 2022 Sep 2;101(35):e30144. doi: 10.1097/MD.0000000000030144 (PMC9439841; doi:10.1097/MD.0000000000030144)
Supplement: Supplementary file 4 [file medi-101-e30144-s004.pdf]

Supplementary figure S4. Subjects achieving perceptible pain relief

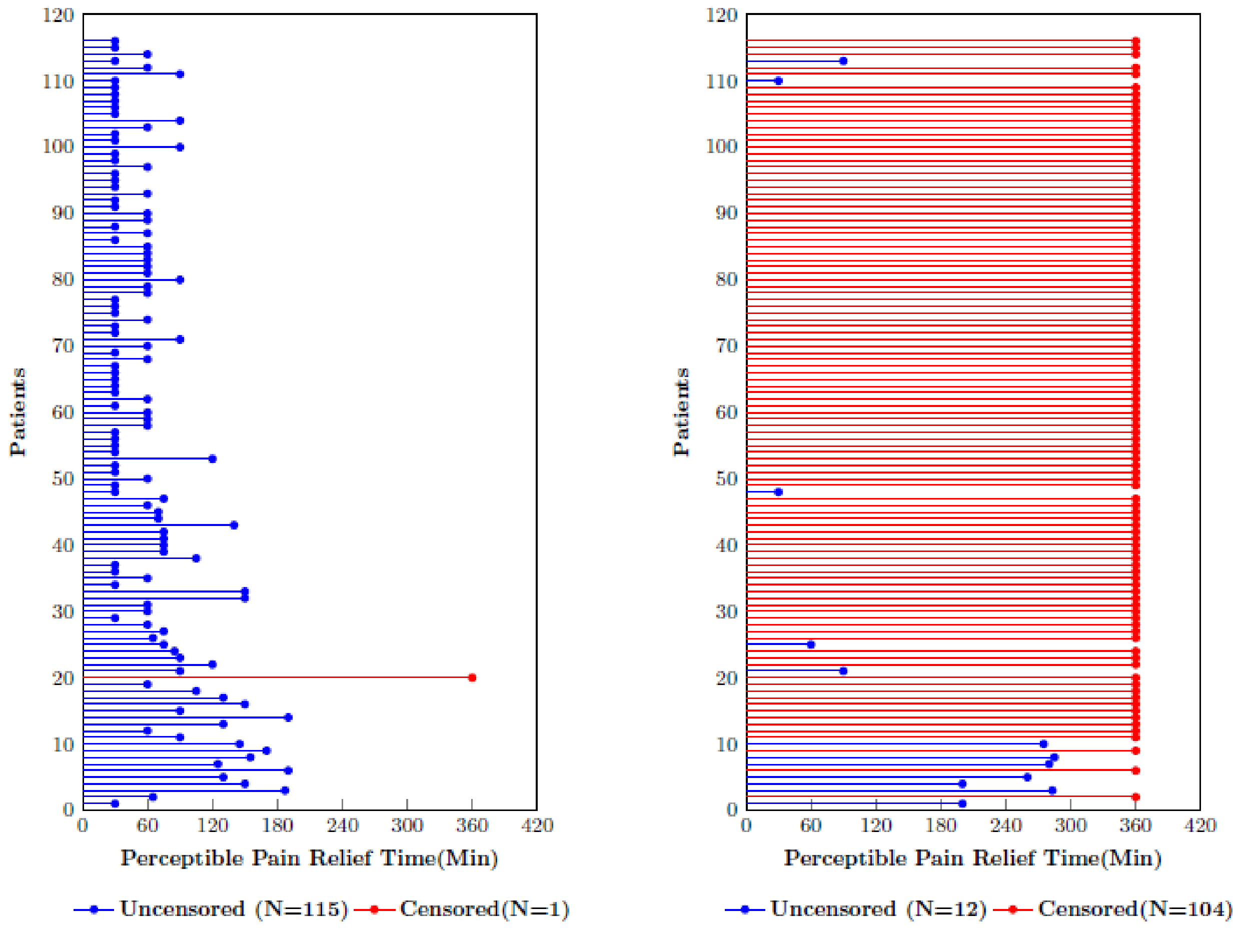

(A) Turmeric-Boswellia Formulation

(B) Placebo

Supplementary figure S5. Subjects achieving Meaningful pain relief

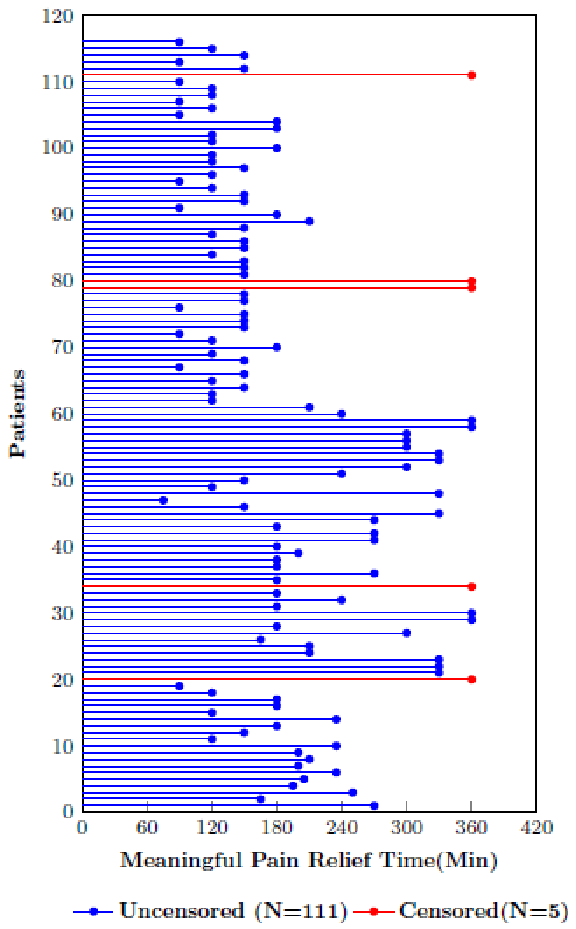

(A) Turmeric-Boswellia Formulation

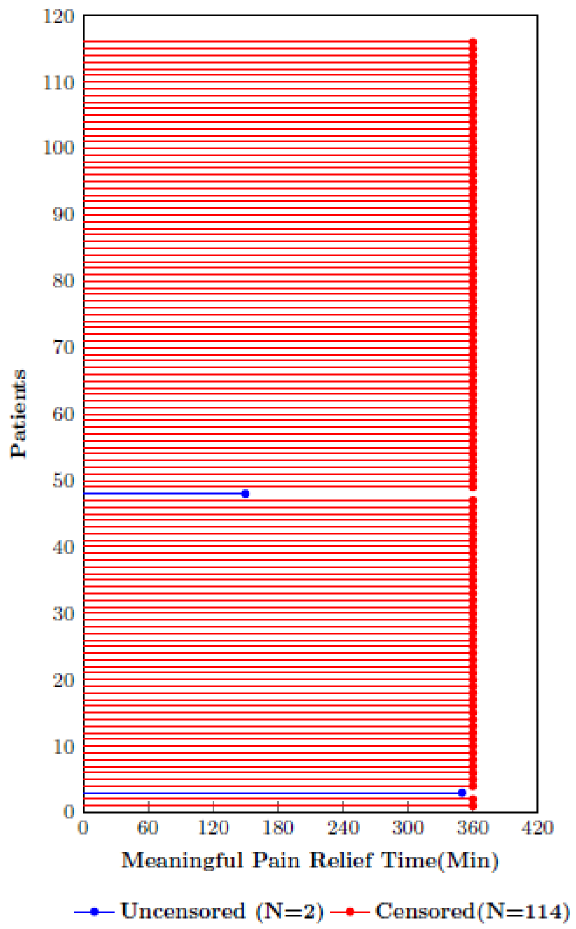

(B) Placebo
