# Supplementary material for: Fast pain relief in exercise-induced acute musculoskeletal pain by turmeric-boswellia formulation: A randomized placebo-controlled double-blinded multicentre study
Source: Medicine (Baltimore). 2022 Sep 2;101(35):e30144. doi: 10.1097/MD.0000000000030144 (PMC9439841; doi:10.1097/MD.0000000000030144)
Supplement: Supplementary file 5 [file medi-101-e30144-s005.pdf]

Supplementary table S3. Kaplan-Meier Product-Limit Survival Analysis for perceptible pain relief in Placebo group							
Event Time	Cumulative Survival	Standard Error	Lower 95% Confidence Limit	Upper 95% Confidence Limit	At Risk	Count	Total Events
30.0	0.9828	0.0121	0.9591	1.0000	116	2	2
60.0	0.9741	0.0147	0.9453	1.0000	114	1	3
90.0	0.9569	0.0189	0.9199	0.9939	113	2	5
200.0	0.9397	0.0221	0.8963	0.9830	111	2	7
260.0	0.9310	0.0235	0.8849	0.9771	109	1	8
275.0	0.9224	0.0248	0.8737	0.9711	108	1	9
280.0	0.9138	0.0261	0.8627	0.9649	107	1	10
283.0	0.9052	0.0272	0.8519	0.9585	106	1	11
285.0	0.8966	0.0283	0.8411	0.9520	105	1	12
360.0+					104		

Supplementary table S4. Kaplan-Meier Product-Limit Survival Analysis for perceptible pain relief in Turmeric-Boswellia formulation (TBF) group							
Event Time	Cumulative Survival	Standard Error	Lower 95% Confidence Limit	Upper 95% Confidence Limit	At Risk	Count	Total Events
30.0	0.6121	0.0452	0.5234	0.7007	116	45	45
60.0	0.3534	0.0444	0.2665	0.4404	71	30	75
65.0	0.3362	0.0439	0.2502	0.4222	41	2	77
70.0	0.3190	0.0433	0.2341	0.4038	39	2	79
75.0	0.2586	0.0407	0.1789	0.3383	37	7	86
85.0	0.2500	0.0402	0.1712	0.3288	30	1	87
90.0	0.1724	0.0351	0.1037	0.2412	29	9	96
105.0	0.1552	0.0336	0.0893	0.2211	20	2	98
120.0	0.1379	0.0320	0.0752	0.2007	18	2	100
125.0	0.1293	0.0312	0.0682	0.1904	16	1	101
130.0	0.1034	0.0283	0.0480	0.1589	15	3	104
140.0	0.0948	0.0272	0.0415	0.1481	12	1	105
145.0	0.0862	0.0261	0.0351	0.1373	11	1	106
150.0	0.0517	0.0206	0.0114	0.0920	10	4	110
155.0	0.0431	0.0189	0.0061	0.0801	6	1	111
170.0	0.0345	0.0169	0.0013	0.0677	5	1	112
187.0	0.0259	0.0147	0.0000	0.0547	4	1	113
190.0	0.0086	0.0086	0.0000	0.0254	3	2	115
360.0+					1		

Supplementary table S5. Kaplan-Meier Product-Limit Survival Analysis for meaningful pain relief in Placebo group							
Event Time	Cumulative Survival	Standard Error	Lower 95% Confidence Limit	Upper 95% Confidence Limit	At Risk	Count	Total Events
150.0	0.9914	0.0086	0.9746	1.0000	116	1	1
350.0	0.9828	0.0121	0.9591	1.0000	115	1	2
360.0+					114		

Formatted: Font color: Auto

Formatted: Normal, Left, Tab stops: Not at 1.51" + 2.52" + 3.52" + 4.53" + 5.24" + 5.89" + 6.55"

Supplementary table S6. Kaplan-Meier Product-Limit Survival Analysis for meaningful pain relief in Turmeric-Boswellia formulation (TBF) group							
Event Time	Cumulative Survival	Standard Error	Lower 95% Confidence Limit	Upper 95% Confidence Limit	At Risk	Count	Total Events
75.0	0.9914	0.0086	0.9746	1.0000	116	1	1
90.0	0.8966	0.0283	0.8411	0.9520	115	11	12
120.0	0.7155	0.0419	0.6334	0.7976	104	21	33
150.0	0.5259	0.0464	0.4350	0.6167	83	22	55
165.0	0.5086	0.0464	0.4176	0.5996	61	2	57
180.0	0.3707	0.0448	0.2828	0.4586	59	16	73
195.0	0.3621	0.0446	0.2746	0.4495	43	1	74
200.0	0.3362	0.0439	0.2502	0.4222	42	3	77
205.0	0.3276	0.0436	0.2422	0.4130	39	1	78
210.0	0.2845	0.0419	0.2024	0.3666	38	5	83
235.0	0.2586	0.0407	0.1789	0.3383	33	3	86
240.0	0.2328	0.0392	0.1559	0.3097	30	3	89
250.0	0.2241	0.0387	0.1483	0.3000	27	1	90
270.0	0.1810	0.0358	0.1110	0.2511	26	5	95
300.0	0.1379	0.0320	0.0752	0.2007	21	5	100
330.0	0.0776	0.0248	0.0289	0.1263	16	7	107
360.0	0.0431	0.0189	0.0061	0.0801	9	4	111
360.0+					5		

Formatted: Font color: Auto

Formatted: Normal, Left, Tab stops: Not at 1.51" + 2.52" + 3.52" + 4.53" + 5.24" + 5.89" + 6.55"
