# Supplementary material for: Fast pain relief in exercise-induced acute musculoskeletal pain by turmeric-boswellia formulation: A randomized placebo-controlled double-blinded multicentre study
Source: Medicine (Baltimore). 2022 Sep 2;101(35):e30144. doi: 10.1097/MD.0000000000030144 (PMC9439841; doi:10.1097/MD.0000000000030144)
Supplement: Supplementary file 6 [file medi-101-e30144-s006.pdf]

Supplementary table S7: Analysis of survival probability between Turmeric-Boswellia formulation (TBF) and placebo groups						
Statistical test	Perceptible Pain Relief			Meaningful Pain Relief		
	Chi-Square	df	p-value	Chi-Square	df	p-value
Logrank	250.513	1.00	<0.001	240.89	1.00	<0.001
Gehan-Wilcoxon	197.3	1.00	<0.001	200.60	1.00	<0.001
Tarone-Ware	223.191	1.00	<0.001	220.74	1.00	<0.001

Supplementary table S8: Log Rank Test Hazard Ratio (Symptom Resolution Ratio) for Perceptible and meaningful pain relief										
Group Ratio		Sample Size (n)	Observed Events (TBF/P)	Expected events (TBF/P)	Hazard rate (TBF/P)	Cox-Mantel Hazard ratio	95%CI (Hazard ratio)		Chi-Square	P value
							Lower	Upper		
TBFt /Placebo	Perceptible Pain Relief	116/ 116	115/12	42.46/84.54	2.71/0.14	19.08	13.20	27.59	186.19	<0.001
	Meaningful Pain Relief	116/| 116	111/2	38.58/74.42	2.88/0.03	107.06	72.57	157.95	206.43	<0.001
TBF/P = Turmeric-Boswellia formulation/Placebo ratio										

Supplementary table S9. COX Regression analysis for symptom resolution rate of Turmeric-Boswellia formulation (TBF) group compared to placebo							
		Coefficient (b)	SE	SRR (e^b)	P value	95% CI (RR)	
						Lower	Upper
Perceptible Pain Relief	NRS at Rest	-0.323	0.079	0.724	<0.001	0.620	0.846
	TBF	4.426	0.383	83.566	<0.001	39.460	176.970
Meaningful Pain Relief	NRS at Rest	-0.446	0.091	0.640	<0.001	0.536	0.766
	TBF	5.606	0.727	272.116	<0.001	65.469	1131.027

SRR: Symptom Resolution Ratio
SE = standard error of coefficient
e^b = exponential of coefficient
RR = Resolution rate
