# Supplementary material for: Fast pain relief in exercise-induced acute musculoskeletal pain by turmeric-boswellia formulation: A randomized placebo-controlled double-blinded multicentre study
Source: Medicine (Baltimore). 2022 Sep 2;101(35):e30144. doi: 10.1097/MD.0000000000030144 (PMC9439841; doi:10.1097/MD.0000000000030144)

Supplementary figure S6. Schoenfield Plot comparing NRS_{rest} with PPR

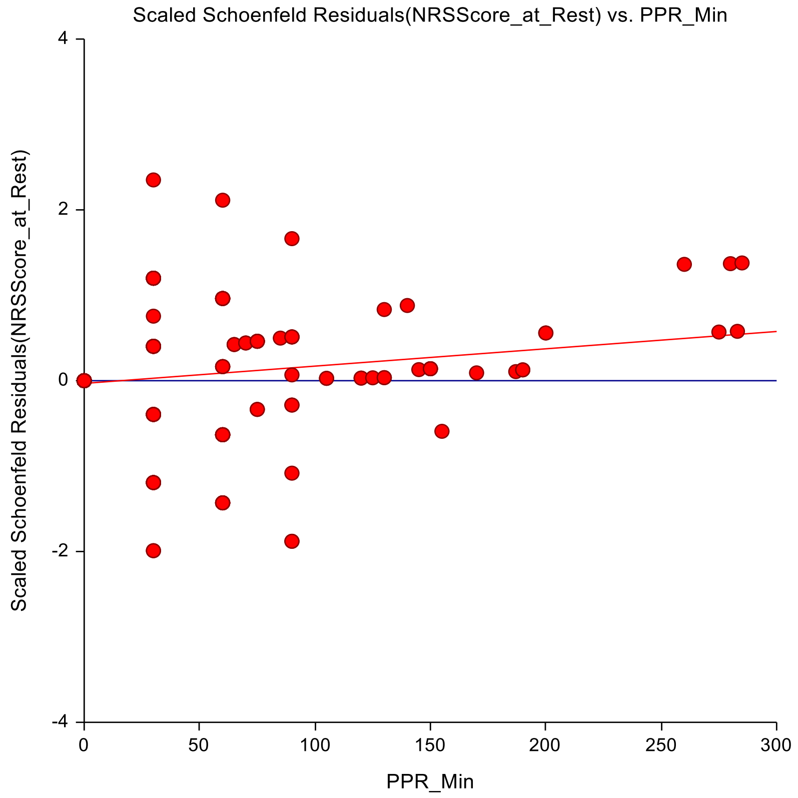

Supplementary figure S7. Schoenfield Plot comparing NRS_{rest} with MPR

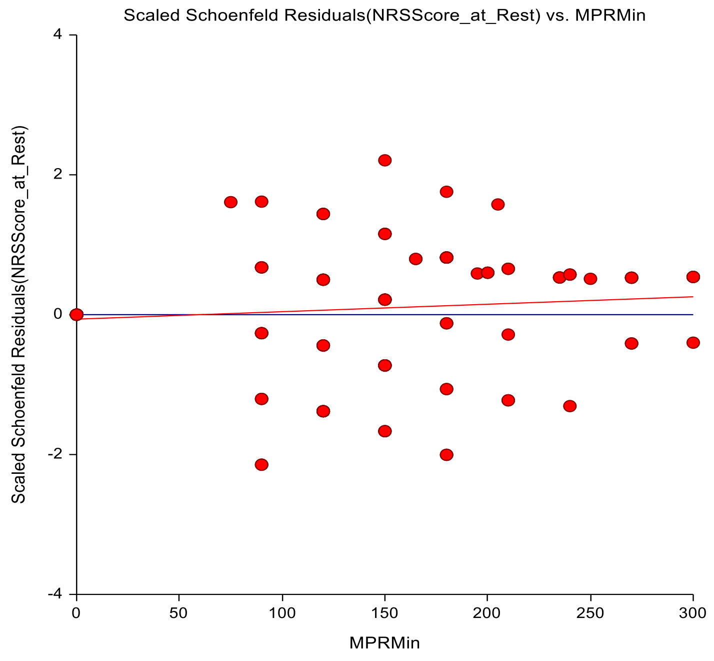

Supplement: Supplementary file 7 [file medi-101-e30144-s007.pdf]
